# Increased pulmonary serotonin transporter in patients with chronic obstructive pulmonary disease who developed pulmonary hypertension

**DOI:** 10.1007/s00259-020-05056-7

**Published:** 2020-10-03

**Authors:** Armin Frille, Michael Rullmann, Georg-Alexander Becker, Marianne Patt, Julia Luthardt, Solveig Tiepolt, Hubert Wirtz, Osama Sabri, Swen Hesse, Hans-Juergen Seyfarth

**Affiliations:** 1 Department of Respiratory Medicine, University Hospital Leipzig, Liebigstrasse 20, 04103 Leipzig, Germany; 2 Integrated Research and Treatment Center (IFB) Adiposity Diseases, University Medical Center Leipzig, 04013 Leipzig, Germany; 3 Department of Nuclear Medicine, University Hospital Leipzig, 04103 Leipzig, Germany

**Keywords:** Serotonin transporter, Positron emission tomography, Computed tomography, Chronic obstructive pulmonary disease, Pulmonary hypertension

## Abstract

**Purpose:**

Pulmonary hypertension (PH) is characterized by a progressive remodelling of the pulmonary vasculature resulting in right heart failure and eventually death. The serotonin transporter (SERT) may be involved in the pathogenesis of PH in patients with chronic-obstructive pulmonary disease (COPD). This study investigated for the first time the SERT in vivo availability in the lungs of patients with COPD and PH (COPD+PH).

**Methods:**

SERT availability was assessed using SERT-selective [^11^C]DASB and positron emission tomography/computed tomography (PET/CT) with dynamic acquisition over 30 min in 4 groups of 5 participants each: COPD, COPD+PH, pulmonary arterial hypertension, and a healthy control (HC). Time activity curves were generated based on a volume of interest within the middle lobe. Tissue-to-blood concentration ratios after 25 to 30 min (TTBR_25–30_) served as receptor parameter for group comparison and were corrected for lung tissue attenuation. Participants underwent comprehensive pulmonary workup. Statistical analysis included group comparisons and correlation analysis.

**Results:**

[^11^C]DASB uptake peak values did not differ among the cohorts after adjusting for lung tissue attenuation, suggesting equal radiotracer delivery. Both the COPD and COPD+PH cohort showed significantly lower TTBR_25–30_ values after correction for lung attenuation than HC. Attenuation corrected TTBR_25–30_ values were significantly higher in the COPD+PH cohort than those in the COPD cohort and higher in non-smokers than in smokers. They positively correlated with invasively measured severity of PH and inversely with airflow limitation and emphysema. Considering all COPD patients ± PH, they positively correlated with right heart strain (NT-proBNP).

**Conclusion:**

By applying [^11^C]DASB and PET/CT, semiquantitative measures of SERT availability are demonstrated in the lung vasculature of patients with COPD and/or PH. COPD patients who developed PH show increased pulmonary [^11^C]DASB uptake compared to COPD patients without PH indicating an implication of pulmonary SERT in the development of PH in COPD patients.

**Electronic supplementary material:**

The online version of this article (10.1007/s00259-020-05056-7) contains supplementary material, which is available to authorized users.

## Introduction

Pulmonary hypertension (PH) is a hemodynamic disorder that affects both the respiratory and cardiovascular system and may cause multiple clinical conditions [1]. According to the current guidelines, PH is defined as an increase in mean pulmonary arterial pressure (PAPm) ≥ 25 mmHg at rest as assessed by right heart catheterization (RHC) [1]. It is characterized by a vasculopathy of the small pulmonary arteries that comprises vasoconstriction and proliferation in all layers of the vessel wall as well as fibrosis and inflammation. It may be complicated by right failure and eventually death. The clinical classification of PH categorizes multiple clinical conditions into five groups: Briefly, PH can be due to (1) pulmonary arterial hypertension (PAH) including idiopathic, familial, drug, and toxin-induced and associated forms, (2) left heart diseases, (3) lung diseases and/or hypoxia, for example, chronic obstructive lung diseases (COPD) and interstitial lung diseases, (4) chronic thromboembolic PH (CTEPH), and (5) unclear multifactorial mechanisms (hematologic, systemic, or metabolic disorders).

Thereof, left heart diseases and lung diseases and/or hypoxia are the most prevalent clinical conditions in PH. The presence of PH in COPD patients is a strong predictor of mortality in COPD [2]. Pathophysiologically, chronic inflammation response due to chronic inhalation of cigarette smoke or other noxious particles induces airway limitation and irreversible parenchymal lung tissue destruction resulting in emphysema, as a hallmark of advanced stage of COPD [3]. As a consequence thereof, pulmonary vascular bed becomes rarefied affecting pulmonary perfusion. The resulting alveolar hypoxia additionally causes hypoxic pulmonary vasoconstriction (HPV), which altogether leads to the development of PH in COPD (COPD+PH).

**Fig. 1:**
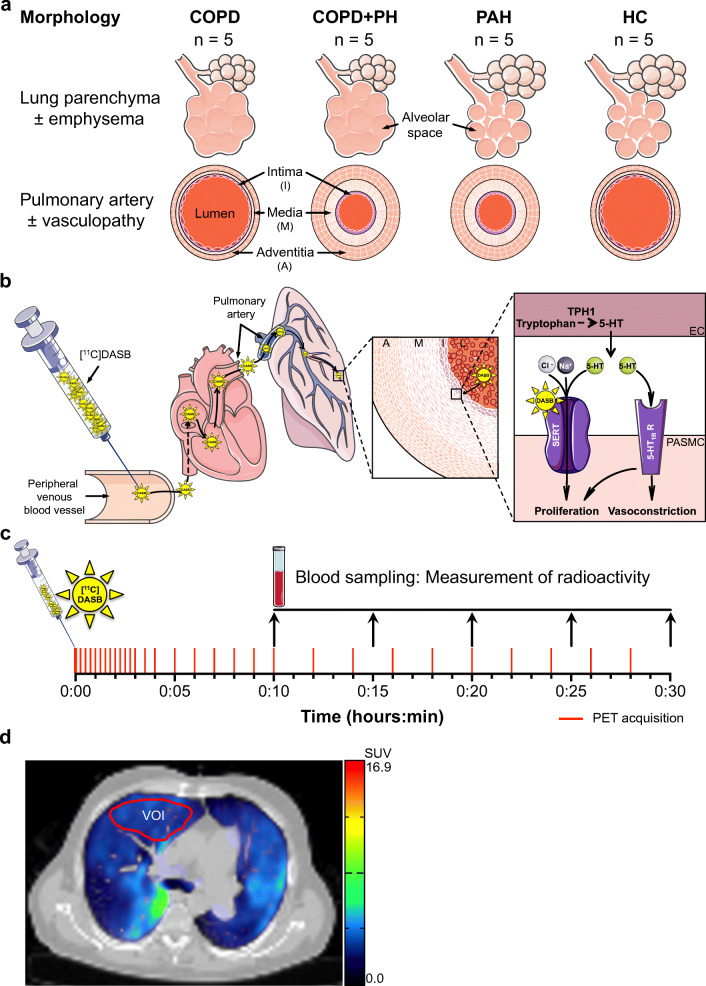
Artwork showing study design and study procedures. (a) Description of cohort’s characteristics (± emphysema, ± vasculopathy). (b) Pathway of [^11^C]DASB through the vascular system with binding to serotonin transporter (SERT) on the pulmonary artery smooth muscle cell (PASMC) surface. (c) Time points of PET acquisitions and blood sampling after [^11^C]DASB administration (d) Representative coregistered transversal PET/CT image of a patient with COPD and PH showing a manually and click-wise selected volume of interest (VOI) in the middle lobe of the lung. On right side of the PET/CT image, a color scale shows the range of SUV values. *5-HT* 5-hydroxtryptamine, *A* adventitia, *Cl*^*-*^ chloride ion, *COPD* chronic obstructive pulmonary disease, *EC* endothelial cell, *HC* healthy control, *I* intima, *L* lumen, *n* number of participants, *Na*^*+*^ sodium ion, *M* media, *min* minute, *PAH* pulmonary arterial hypertension, *PASMC* pulmonary arterial smooth muscle cells, *PH* pulmonary hypertension, *SUV* standardized uptake value, *TPH1* tryptophan hydroxylase 1

A role of serotonin, or 5-hydroxytryptamine (5-HT), in the development of PH has been proposed for many decades due to serotonin’s vasoconstrictive and proliferative properties leading to the so-called serotonin hypothesis of PH [4]. Evidence suggests that pulmonary endothelial cells from PAH patients overexpress tryptophan hydroxylase 1 (TPH1) leading to increased endothelial serotonin synthesis and secretion towards pulmonary arterial smooth muscle cells (PASMC) [5]. Serotonin induces relevant vasoconstriction in the pulmonary arteries via its receptors (e.g., 5-HT_1B_) and proliferation of PASMCs via both its receptors and transporters (Fig. 1), subsequently contributing to the development or aggravation of PH [6–8]. Increased serotonin signalling has been implicated in the development of PAH after the use of antidepressants in pregnant women giving birth to new-borns with persistent PH and appetite suppressants as they act as selective serotonin reuptake inhibitors (SSRI) or serotonin receptor agonists [1]. Moreover, in PAH patients, incident SSRI use was associated with increased mortality and a greater risk of clinical worsening [9].

The serotonin transporter (SERT) is a Na^+^/Cl^−^-coupled symporter for the biogenic amine serotonin and functions to reduce extracellular levels of serotonin, as it does on the presynaptic nerve terminals in the central nervous system [10]. The lung has been shown to play a major role in the physiologic removal of circulating serotonin through pulmonary SERT [11–13]. Increased expression or activity of SERT in the PASMC has been observed in patients with PAH and COPD+PH [6, 14]. Consistent with this observation is the fact that hypoxic SERT knockout mice develop less severe PH and vascular remodelling than the wild-type counterpart [15] emphasizing the role of SERT in the pathophysiology of PH under hypoxic conditions, such as in COPD patients.

Positron emission tomography (PET) studies using an ^11^C-labelled antidepressant directed to target SERT found high accumulation in the lung [16]. This suggests that the lung functions as a reservoir for antidepressants via binding to SERT. The SERT-selective radioligand [^11^C]DASB, which is a sulfanyl-benzonitrile derivate, is clinically used by means of PET and computed tomography (PET/CT) imaging in the fields of schizophrenia, epilepsy, and depression [17]. Moreover, [^11^C]DASB and PET imaging also demonstrated pulmonary SERT availability in humans [18], and the intake of SERT-selective paroxetine reduced the lung uptake of a radioligand selective for monoamine transporter and SERT ([^123^I]FP-CIT) as compared to placebo intake as shown by scintigraphy [19].

In here, we carried out a first-in-human pilot study using semiquantitative uptake measures of the SERT-selective radioligand [^11^C]DASB to assess whether the serotonin pathway in the lung is pathophysiologically relevant for the development of PH in patients with COPD. To this end, we hypothesized that COPD patients who developed PH (COPD+PH) show increased pulmonary SERT availability compared with COPD patients without PH.

## Methods

### Study design and subjects

We performed a prospective pilot study of 15 patients with COPD, COPD+PH, or PAH, and five healthy controls (HC) (Fig. 1(a)). We selected these participants from the inpatient and outpatient clinic of our Department of Respiratory Medicine. Inclusion criteria consisted of parameters from the respiratory workup defining advanced stage of COPD and/or PH, respectively. Participants not meeting the criteria for advanced disease stage were not included. As a control group, we recruited healthy volunteers, who were matched to age and were non-smokers.

### Clinical, laboratory, and hemodynamic assessment

The diagnoses for COPD, COPD+PH, and PH were established according the current clinical guidelines [1, 20]. The respiratory workup for participants comprised pulmonary function test (PFT) including spirometry and body plethysmography, 6-min walking distance (6MWD) including the Borg score for rating dyspnoea (0 points: no dyspnoea; 10 points: maximal dyspnoea), capillary blood gas analysis (BGA), diffusing capacity of the lung for carbon monoxide (DLCO), serum levels of N-terminal pro-brain natriuretic peptide (NT-proBNP), as well as the calculation of the oxygenation ratio (arterial oxygen tension [PaO_2_]/inspiratory oxygen fraction [FiO_2_]), and alveolar-arterial gradient (A-aO_2_). Disease severity and risk of death estimates for COPD patients were assessed using the multidimensional BODE score that includes body mass index (B), airflow obstruction (O), dyspnoea (D), and exercise capacity (E) [21]. Right heart catheterization (RHC) provided invasively measured parameters on mean pulmonary arterial pressure (PAPm), pulmonary vascular resistance (PVR), and cardiac index (CI), establishing the diagnosis of PH and determining its severity. Severity of PH was clinically evaluated using the World Health Organization functional class (WHO-FC) [22]. Care has been taken that none of the participants were under SSRI or monoamine oxidase (MAO) inhibitors therapy during study enrolment.

### [^11^C]DASB PET/CT

Pulmonary SERT availability was assessed after intravenous injection of SERT-selective [^11^C] 3-amino-4-(2-dimethylaminomethylphenylsulfanyl)-benzonitrile ([^11^C]DASB) with averaged 495 ± 6 MBq using an integrated PET/CT scanner (Biograph 16 PET/CT Scanner [Siemens Medical Solutions, Erlangen, Germany]). The dynamic acquisition was performed over 30 minutes (min) and comprised 30 measuring points (12 × 15 s [seconds], 2 × 30 s, 6 × 60 s, 10 × 120 s). A low-dose CT of the lung was performed in each subject for anatomic coregistration and attenuation correction. The radioactivity in blood was measured at 10, 15, 20, 25, and 30-min post injection (p.i.) in each participant using a Cobra gamma counter (Packard Instrument Company, Meriden, CT, USA) and decay was corrected to the start time of the PET/CT scan (Fig. 1(b, c)).

### Image analysis

Time activity curves (TACs) were generated from each of the 30 measuring points based on a volume of interest (VOI), which were manually defined within the middle lobe of the lung, averaging 14.0 ± 4.7 mm^3^ without intergroup differences (Fig. 1(d)). Standardized uptake values (SUV) were computed from tracer activity measured with PET according to SUV = tracer-activity-in-tissue/(injected-dose/body-weight). Both the maximum standardized uptake value (SUV_max_) after 0 to 3 min and the SUV after 25 to 30 min (SUV_25-30_) served as model-free parameter for group comparison. Tissue-to-blood (TTBR) and tissue-to-plasma (TTPR) concentration ratios were calculated by means of the averaged activity of the last two blood or plasma samples (25–30 min p.i.), respectively.

### Statistical analysis

Data were compiled and their distributions were estimated (histogram analysis, skewness, kurtosis, Shapiro-Wilk test). Group differences were calculated using the Student’s *t* test or Mann-Whitney *U* test for comparison of 2 groups and one-way analysis of variance (ANOVA) followed by Tukey’s post hoc correction for comparison of > 2 groups. Analysis of covariance (ANCOVA) was performed to adjust the TTBR and TTPR values for the mean lung tissue attenuation of the middle lobe. The calculation of partial Spearman rank correlation coefficient between TTBR_25–30_ (independent variable) and clinical or hemodynamic parameters (dependent variables) was used to adjust for lung tissue attenuation (control variable). Statistical significance was accepted at a level of a two-sided *P* < 0.05. Results are expressed as mean ± standard deviation (SD) or 95% confidence interval (CI). Data analysis, calculation, and preparation of figures were conducted using the software package GraphPad Prism (v8.3.0 for macOS, La Jolla, California, USA), SPSS (v25.0, IBM Corporation, Chicago, IL, USA), Matlab (v7.13, The MathWorks Inc., Natick, MA, USA), and R: A Language and Environment for Statistical Computing (v3.4, R Foundation for Statistical Computing, Vienna, Austria, 2017, http://www.R-project.org). Figure 1 contains modified graphic content provided by Servier Medical Art by Servier (https://smart.servier.com) licensed under a Creative Commons Attribution 3.0 unported license (CC BY 3.0).

## Results

### Characteristics of subjects

A coregistered [^11^C]DASB using PET/CT with dynamic acquisition over 30 min and a respiratory workup was performed in 19 participants (1 participant was not measured by PET/CT for technical reasons). In addition, participants underwent hemodynamic assessment by means of RHC, except for the HC. All PAH patients received a drug combination therapy consisting of an endothelin receptor antagonist (ERA) plus a phosphodiesterase type 5 inhibitor (PDE-5i) (3/5) or a guanylate cyclase stimulator (2/5) and an inhaled prostacyclin analogue (1/5). In COPD+PH cohort, 3/5 participants received a specific drug therapy, of which 2 were on monotherapy (PDE-5i) and 1 on combination therapy (PDE-5i plus ERA).

Table 1 gives an overview on the clinical, hemodynamic, laboratory, and radiologic characteristics of the participants. Age and body mass index (BMI) were equally distributed among the 4 cohorts. All COPD patients with or without PH exhibited severe airflow limitation while COPD patients without PH significantly expressed a higher GOLD stage (*P* < 0.05) and higher BODE score (*P* < 0.05) than COPD+PH patients (Supplementary Fig. 1). COPD patients showed stronger airflow limitation (lower FEV_1_ values, higher total airway resistance [*R*_tot_] values), higher ratio values of residual volume/total lung capacity (RV/TLC) as a surrogate for emphysema and poorer diffusing capacity (lower DLCO values) as compared to the PAH and HC cohorts. Additional analysis of clinical and hemodynamic characteristics of COPD patients with or without PH is shown in the Supplementary Fig. 2.

**Table 1:** Characteristics of participants

Characteristic	COPD	COPD+PH	PAH	HC
Participants, *n*	5	5	5	5
Sex, male/female	4/1	4/1	0/5	2/3
Age (years)	57.2 ± 2.8	55.8 ± 9.9	57.0 ± 2.8	56.2 ± 8.0
BMI (kg × m^−2^)	25.5 ± 3.2	21.6 ± 2.3	24.6 ± 4.7	26.3 ± 4.1
Smokers, *n*, pys	5, 50 (30–60)	3, 30 (20–50)	0	0
BODE score^‡^	7 (7–9)	5 (3–7)	-	-
WHO-FC	-	3 (3–4)	3 (3–4)	-
Pulmonary function test				
FEV_1_ (% predicted)^‡^	22.1 ± 4.1	46.3 ± 17.3	79.6 ± 16.0	98.7 ± 12.8
COPD stage	4	3 (2–4)	-	-
*R*_tot_ (% predicted)^‡^	508.3 ± 285.3	168.2 ± 87.0	81.2 ± 26.9	60.2 ± 19.9
RV/TLC (%)^‡^	57.7 ± 2.7	62.5 ± 10.8	36.2 ± 8.1	44.4 ± 11.3
DLCO (% predicted)^‡^	16.9± 13.5	18.9 ± 6.3	55.2 ± 19.8	-
Blood gas analysis*				-
PaCO_2_ (mm Hg)^‡^	44.3 ± 2.1	33.1 ± 5.5	29.4 ± 1.7	
PaO_2_ (mm Hg)	56.3 ± 4.8	52.8 ± 9.7	68.6 ± 16.5	
PaO_2_/FiO_2_ (mm Hg)	231.1 ± 46.5	264.0 ± 51.0	323.8 ± 75.8	
SaO_2_ (%)	89.4 ± 2.1	91.2 ± 3.5	88.3 ± 11.2	
A-aO_2_ (mm Hg)	35.9 ± 3.3	49.5 ± 11.4	43.9 ± 21.2	
Exercise capacity				-
6MWD (m)^‡^	205.6 ± 101.3	341.0 ± 72.5	424.0 ± 128.4	
Need for O_2_ at rest, *n* (%)	5 (100)	4 (80)	1 (20)	
O_2_ flow (L × min^−1^)^†^	4 (2–10)	4 (0–5)	0 (0–4)	
Borg score^‡^	7 (5–9)	7 (5–7)	5 (4–6)	
Hemodynamic parameters				-
PAPm (mm Hg)^‡^	23.8 ± 2.1	51.2 ± 5.5	61.8 ± 13.9	
CI (L × min^−1^ × m^−2^)^‡^	3.2 ± 0.5	1.9 ± 0.5	2.3 ± 0.3	
PVR (Wood unit)^‡^	1.8 ± 0.5	11.8 ± 2.2	14.6 ± 3.2	
Biochemical parameter				-
NT-proBNP (ng × L^−1^)^‡^	83 ± 60	2,085 ± 924	1,516 ± 1,619	
Radiologic parameter				
Lung tissue attenuation (HU)^‡^	− 882.2 ± 24.3	− 828.0 ± 45.1	− 771.3 ± 100.3	− 780.0 ± 44.3

Data are shown as mean ± SD or median with range

*Measured at rest without or with lowest as tolerable nasal oxygen flow rate

^†^Via nasal cannula

^‡^Significant differences (*P* < 0.05) using ANOVA for > 2 groups or Mann-Whitney *U* test for 2 groups

*6MWD* 6-min walking distance, *A-aO*_*2*_ alveolar–arterial gradient, *BODE* risk of death estimate for COPD patients, *CI* cardiac index, *COPD* chronic obstructive pulmonary disease, *DLCO* diffusion capacity for carbon monoxide after a single breath, *FEV*_*1*_ forced expiratory volume in 1 s, *FiO*_*2*_ inspiratory oxygen fraction, *FVC* forced vital capacity, *HC* healthy control, *HU* Hounsfield unit, *n* number of participants, *NT-proBNP* N-terminal pro-brain natriuretic peptide, *PaCO*_*2*_ arterial carbon dioxide tension, *PAH* pulmonary arterial hypertension, *PaO*_*2*_ arterial oxygen tension, *PAPm* mean pulmonary arterial pressure, *PH* pulmonary hypertension, *PVR* pulmonary vascular resistance, *R*_*tot*_ total airway resistance, *RV* residual volume, *SaO*_*2*_ arterial oxygen saturation, *TLC* total lung capacity, *WHO-FC* functional classification of pulmonary hypertension according to the World Health Organization

### Pulmonary SERT was quantifiable using [^11^C]DASB PET/CT

**Fig. 2:**
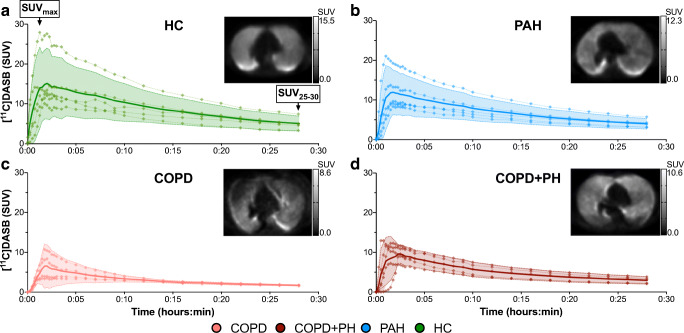
Time activity curves of pulmonary [^11^C]DASB uptake measured within the middle lobe. The time course of [^11^C]DASB uptake is made of the individual measuring points of each participant and is overlaid with mean values ± 95% confidence interval. The labelling of the axes applies to all panels within the figure, respectively. Each panel contains a representative transversal PET image including the middle lobe at 25- to 30-min post injection, and a black-white scale showing the range of SUV values. *CI* confidence interval, *COPD* chronic obstructive pulmonary disease, *HC* healthy control, *PAH* pulmonary arterial hypertension, *PH* pulmonary hypertension, *SUV* standardized uptake value, *VOI* volume of interest

Pulmonary uptake of SERT-selective [^11^C]DASB was quantified using PET/CT in all cohorts investigated (Fig. 2). TACs of all cohorts show initial peak of SUV, designating the pulmonary perfusion and radiotracer delivery and a subsequent decline in SUV for the rest of the acquisition time.

### Pulmonary perfusion in COPD did not affect pulmonary [^11^C]DASB uptake

**Fig. 3:**
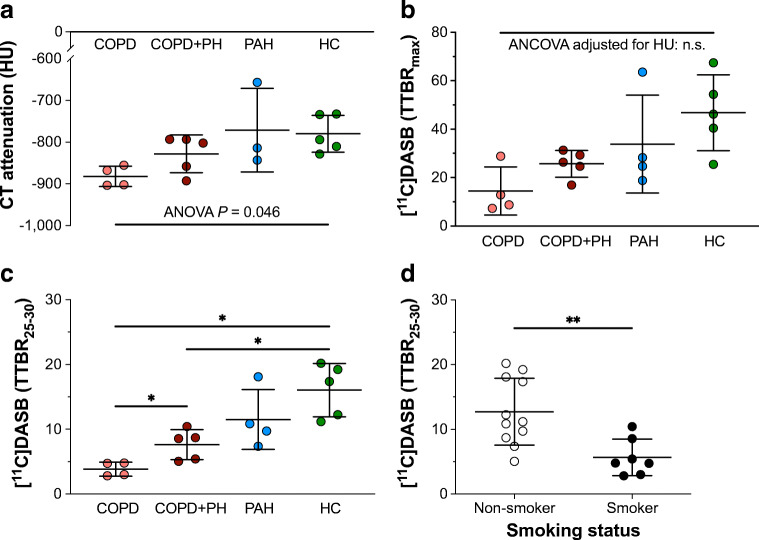
Group comparisons of lung tissue attenuation and pulmonary [^11^C]DASB uptake between the cohorts. (a) Distribution of lung tissue attenuation values among the cohorts measured in HU derived from CT imaging. Overall differences were calculated by means of ANOVA. (b) Group comparison of maximum pulmonary [^11^C]DASB uptake between the cohorts: After correction for blood activity (TTBR_max_) and for attenuation values by means of ANCOVA, the maximum pulmonary [^11^C]DASB uptake, i.e., pulmonary blood flow, did not differ between the cohorts. (c) Group comparisons of [^11^C]DASB uptake between the cohorts included the correction for blood activity (TTBR_25–30_) and for lung tissue attenuation by means of ANCOVA. (d) Group differences in TTBR_25–30_ of [^11^C]DASB in relation to smoking status. Statistical significance was accepted at a level of a two-sided *P* < 0.05. Statistically non-significant comparisons are not labelled, *P < 0.05, **P < 0.01. Group differences are shown by mean ± standard deviation. *ANCOVA* analysis of covariance, *ANOVA* analysis of variance, *COPD* chronic obstructive pulmonary disease, *HC* healthy control, *n.s.* not significant, *PAH* pulmonary arterial hypertension, *PH* pulmonary hypertension, *SD* standard deviation, *TTBR* tissue-to-blood concentration ratio

The lung tissue attenuation measured by means of CT among the cohorts only differed in the ANOVA, but not in the post hoc comparisons (Fig. 3(a)). Both TTBR_max_ and TTPR_max_ corrected for lung attenuation (ANCOVA) did not differ between the cohorts (Fig. 3(b)).

### Pulmonary [^11^C]DASB uptake significantly differed between COPD patients with and without PH

Both the COPD and COPD+PH cohort showed significantly lower attenuation corrected TTBR_25–30_ and TTPR_25–30_ values than the HC (Fig. 3(c)). Attenuation corrected TTBR_25−30_ values were significantly higher in COPD+PH than in COPD patients without PH (*P* = 0.038), while using attenuation corrected TTPR_25−30_ values, this group comparison confirmed the trend but did not reach, if only just, the significance level (*P* = 0.054). Both attenuation corrected TTBR_25–30_ and TTPR_25–30_ did not differ between the HC and PAH cohort (*P* = 0.243, *P* = 0.172, respectively). Smokers showing long-term exposure (averaging 43 pack years) were measured significantly lower TTBR_25–30_ values than the non-smokers (5.7 ± 2.8 vs. 12.7 ± 5.2, *P* = 0.005) (Fig. 3(d)).

### Pulmonary [^11^C]DASB uptake significantly correlated with clinical and hemodynamic parameters

**Fig. 4:**
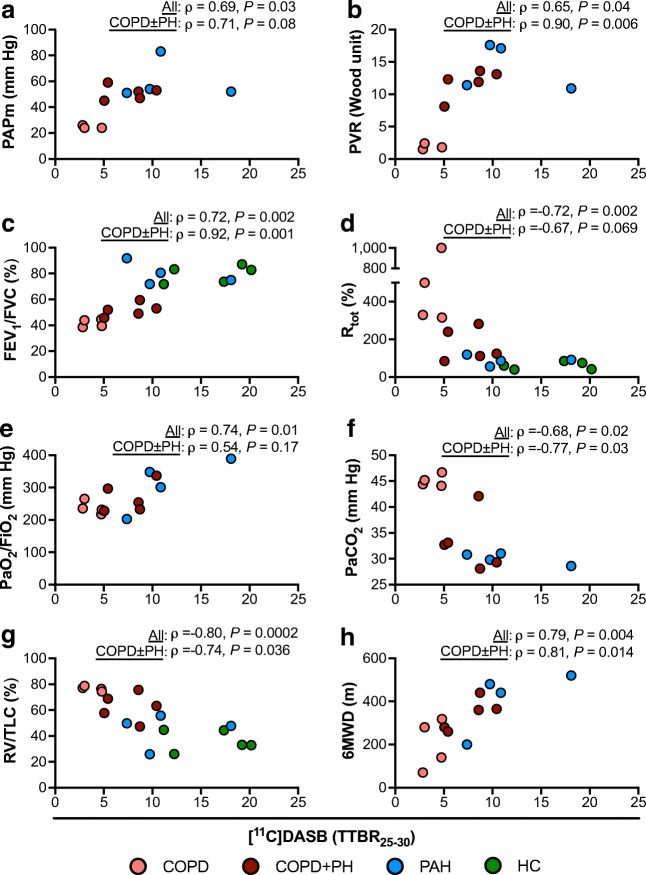
Scatter plots of [^11^C]DASB uptake and hemodynamic (a, b) and clinical parameters (c–h). Partial Spearman rank correlation analysis was performed to correct for lung tissue attenuation. Results are depicted as Spearman rank correlation coefficient rho (*ρ*). Statistical significance was accepted at a two-sided *P* < 0.05. The labelling of the abscissa applies to all panels. *6MWD* 6-min walking distance, *COPD* chronic obstructive pulmonary disease, *FEV*_*1*_ forced expiratory volume in 1 s, *FVC* forced vital capacity, *HC* healthy control, *PaO*_*2*_*/FiO*_*2*_ oxygenation ratio: arterial oxygen tension/inspiratory oxygen fraction, *PAH* pulmonary arterial hypertension, *PAPm* mean pulmonary arterial pressure, *PaCO*_*2*_ arterial oxygen tension, *PH* pulmonary hypertension, *PVR* pulmonary vascular resistance, *RV* residual volume, *TLC* total lung capacity, *TTBR*_*25-30*_ tissue-to-blood concentration ratio after 25 to 30 minutes of [^11^C]DASB administration

Partial Spearman rank correlation analysis of TTBR_25–30_ was performed with clinical and hemodynamic parameters to adjust for lung tissue attenuation (Fig. 4, Table 2). With regards to severity of PH, attenuation corrected TTBR_25–30_ values positively correlated with PAPm and PVR (*ρ* = 0.69 and *ρ* = 0.65, respectively). In terms of severity of COPD, TTBR_25–30_ values that were corrected for lung attenuation positively correlated with FEV_1_/FVC (*ρ* = 0.72), as an indicator of airflow limitation, and inversely with RV/TLC (*ρ* = − 0.80), as an indicator of the degree of pulmonary emphysema. Exercise capacity evaluated by 6MWD positively correlated with TTBR_25–30_ values corrected for lung attenuation (*ρ* = 0.79). Concerning the pulmonary vascular mismatch, attenuation corrected TTBR_25–30_ positively correlated with the oxygenation ratio, i.e., PaO_2_/FiO_2_ (*ρ* = 0.74) and with the diffusing capacity for carbon monoxide, i.e., DLCO (*ρ* = 0.77) and negatively with hypercapnia, i.e., PaCO_2_, measured by BGA (*ρ* = − 0.68).

**Table 2:** Partial correlation of TTBR_25–30_ with pulmonary function and biochemical and hemodynamic parameters with correction for lung tissue attenuation

		Airflow limitation						Emphysema		Gas exchange								Exercise capacity		Right heart strain		Hemodynamic					
		FEV_1_/FVC		FEV_1_		*R*_tot_		RV/TLC		DLCO		PaO_2_/ F_i_O_2_		SaO_2_		PaCO_2_		6MWD		NT-proBNP		PAPm		PVR		CI	
All*	*ρ*		*0.72*		*0.73*	−	0*.71*	−	*0.80*		*0.77*		*0.74*		*0.69*	−	*0.68*		*0.79*		0.07		*0.69*		*0.65*	**−**	0.36
	*P*		0.002		0.001		0.002		0.0002		0.01		0.01		0.02		0.02		0.004		0.85		0.03		0.04		0.31
	*n*		17		17		17		17		11		12		12		12		12		12		11		11		11
COPD ± PH	*ρ*		*0.92*		*0.73*	**−**	0.67	**−**	*0.74*		0.42		0.54		0.45	**−**	*0.77*		*0.81*		*0.83*		0.71		*0.90*	−	*0.92*
	*P*		0.001		0.04		0.069		0.036		0.35		0.17		0.26		0.03		0.014		0.01		0.075		0.006		0.003
	*n*		9		9		9		9		8		9		9		9		9		9		8		8		8

*Taking into account the data available from the 4 cohorts, e.g., there were no measurements of gas exchange and hemodynamic in HC. Partial Spearman rank correlation (*ρ*) of TTBR_25–30_ with pulmonary function and biochemical and hemodynamic parameters was used to correct for lung tissue attenuation of the middle lobe measured by computed tomography between the 4 cohorts (i.e., all) and between the COPD cohort with or without PH (i.e., COPD ± PH). Statistically significant partial correlations were accepted at a level of a two-sided *P* < 0.05 and are reported in italic

*6MWD* 6-min walking distance, *BGA* blood gas analysis, *CI* cardiac index, *DLCO* diffusion capacity for carbon monoxide after a single breath, *FEV*_*1*_ forced expiratory volume in 1 s, *FiO*_*2*_ inspiratory oxygen fraction, *FVC* forced vital capacity, *n* number of participants, *NT-proBNP* N-terminal pro-brain natriuretic peptide, *PaCO*_*2*_ carbon dioxide tension in arterial blood, *PaO*_*2*_ oxygen tension in arterial blood, *PAPm* mean pulmonary arterial pressure, *PFT* pulmonary function test, *PH* pulmonary hypertension, *PVR* pulmonary vascular resistance, *R*_*tot*_ total airway resistance, *RV* residual volume, *TLC* total lung capacity, *TTBR*_*25–30*_ tissue-to-blood concentration ratio of pulmonary [^11^C]DASB uptake

## Discussion

To the best of our knowledge, this is the first prospective trial to study the pulmonary SERT availability in COPD patients having developed PH by using [^11^C]DASB and PET/CT. By applying a semiquantitative approach, we found that [^11^C]DASB was not only measurable in the lung parenchyma of the 4 cohorts tested; its uptake also differed significantly between participants with COPD and with COPD+PH. Pulmonary SERT availability correlated positively with the severity of PH, arterial oxygenation (PaO_2_/FiO_2_), and exercise capacity (6MWD) as well as inversely with airflow limitation, emphysema, and carbon dioxide retention. Furthermore, pulmonary SERT availability was found significantly reduced in long-term smokers compared to non-smokers.

The serotonin hypothesis of PH has been proposed for many decades due to serotonin’s vasoconstrictive and proliferative properties [4]. The lung has been shown to play a major role in the physiological removal of circulating serotonin through the presence of pulmonary SERT [11–13]. The SERT-selective tracer [^11^C]DASB and PET imaging studies successfully demonstrated pulmonary SERT availability in humans [18]. There are other SERT-selective radioligands available for humans as well [23]. Here, we chose to apply [^11^C]DASB because it binds with high affinity and selectivity to the SERT [24, 25], proving to be suitable for the pulmonary SERT quantification [18, 26]. We provide evidence that pulmonary SERT can be visualized and quantified in patients with COPD and/or PH using [^11^C]DASB and PET/CT imaging.

Smoke-induced chronic inflammation response leads to parenchymal lung tissue destruction and emphysema, representing a hallmark of advanced COPD stage [3]. As a consequence thereof, pulmonary vascular bed becomes rarefied which affects the pulmonary perfusion. The middle lobe was selected for [^11^C]DASB uptake analyses since smoke-induced centrilobular emphysema has a typical apical distribution and affects less commonly the middle lobe. Nevertheless, we found significant differences in the variances of lung tissue attenuation values (Fig. 3(a)), which is suggestive of emphysematous and, thus, vascular changes of the lung parenchyma. Likewise, the peak values of [^11^C]DASB uptake corrected for blood radioactivity (TTPR_max_, TTBR_max_), likely representing pulmonary perfusion and radiotracer delivery, significantly varied between the cohorts (Fig. 3(b)). These differences vanished not until TTBR_max_ values were corrected for lung tissue attenuation suggesting that pulmonary [^11^C]DASB uptake was not different between the 4 cohorts.

The physical and biological half-lives of [^11^C]DASB in humans approximately amount to 20 and 51 min, respectively. The mean residence time of the tracer is measured 7.6 min, while less than 10% of injected dose are found in the lungs of HC after 30 min of administration [18]. We carried out [^11^C]DASB uptake analyses between 25 and 30 min p.i. and corrected SUV for both blood and plasma radioactivity (TTPR_25–30_, TTBR_25–30_) as well as for lung tissue attenuation. Thereby, we consider the measurement of pulmonary [^11^C]DASB uptake 25 to 30 min p.i. to be radiotracer-specific for SERT quantification.

It is not possible to apply a compartment model for DASB in the lung. DASB does produce many metabolites [24, 26, 27], which we did not measure in this study, but are yet necessary to obtain an arterial input function for subsequent kinetic modelling. Also, these metabolites are transported into the lung. Thus, the blood and tissue radioactivity measured represents the sum of the parent compound (DASB) and its resulting metabolites, which were not taken into account in this study. The TTBR and TTPR of [^11^C]DASB, however, represent a rough approximation of the distribution volume and therefore receptor density in the lung. Furthermore, the lung has a dual blood supply provided by the pulmonary and bronchial circulation, making compartment model assumptions and kinetic modelling of the [^11^C]DASB and its metabolites very complex. Altogether, these considerations led us to favor a model-free analysis of [^11^C]DASB uptake in the lung by selecting time frames of interest, which were assumed to coincide with the tissue to blood equilibrium of the radiotracer in all subjects [17].

To correct the pulmonary [^11^C]DASB uptake for blood activity, we included the activity measured in both plasma and blood samples. Statistical analysis of the resulting ratios (TTPR, TTBR) showed nearly consistent results.

There was no increase in pulmonary SERT availability measured using [^11^C]DASB in PAH patients compared to HC. This finding contrasts with results from previous investigations that have found an overexpression of SERT in PASMC from PAH patients [5, 6, 28]. In the present study, the PAH cohort consisted of patients with relevant clinical (median WHO-FC III) and hemodynamic impairment (PAPm 61.8 ± 13.9 mm Hg) due to PH. Compared to the PAH patient’s PAPm of the 3 aforementioned studies (62.0 ± 13.0, 62.6 ± 3.9, and 63 ± 11.0 mm Hg, respectively) performing an ex post ANOVA, there was no overall difference in the variances between these 4 studies (*F* = 0.023, overall *P* = 0.99). Thus, the difference in SERT expression or [^11^C]DASB availability cannot be attributed to the severity of PH. Likewise, the PAH and COPD+PH cohort in the present study did not differ in the clinical and hemodynamic impairment as well as in the TTPR_25–30_ values that were corrected for lung attenuation (Table 1).

Notably, COPD and COPD+PH patients who were altogether hypoxemic (SaO_2_, PaO_2_/FiO_2_) and did neither differ in the degree of airway limitation (FEV_1_) nor in the degree of emphysema (RV/TLC) (Table 1, Supplementary Fig. 1), were measured significantly lower TTPR_25–30_ values corrected for lung attenuation than those in the HC. Consistent with this observation is the fact that several rodent models indicate that chronic exposure to hypoxia not only leads to reduced serotonin uptake into the lung [29, 30], but also to downregulation of SERT mRNA or protein expression in PASMC [31–33]. Thus, it may be plausible that hypoxemic COPD patients showed a reduced pulmonary SERT availability in PET/CT using [^11^C]DASB. However, these reports are challenged by other investigations showing that hypoxia increases SERT expression in PASMC, both in 2 rodent models [34, 35] and in 1 human cohort of COPD+PH patients [14]. MacLean and colleagues argued [32] that the variation in the results of these studies might be due to age, sex, or strain differences of the rodents combined with differences in hypoxic exposure and/or resident atmospheric pressures. For instance, only female but not male mice, both overexpressing pulmonary SERT, develop PAH. Their female sex hormone 17β oestradiol is found to induce PAMSC proliferation in the presence of serotonin via stimulating the 5-HT_1B_ receptor [36]. In the present study, there was an uneven gender distribution across the 3 patient cohorts consisting of predominantly male participants in the COPD and COPD+PH cohort (4/5 male, respectively), while exclusively female participants were in the PAH cohort. One cannot rule out the possibility that the gender distribution influenced the SERT availability, which, nevertheless, was balanced among COPD patients with or without PH.

Taken together, since SERT availability in the present study was reduced in hypoxemic COPD patients (Fig. 3(c), Table 1), a hypoxia-induced downregulation of SERT could explain our findings.

Even though COPD patients with or without PH expressed reduced pulmonary SERT availability compared to HC, there was still a significant increase in SERT availability when COPD patients developed PH (Fig. 3(c)). This suggests, that the upregulation of SERT may play a potential role in the development of PH in COPD patients.

In addition to the quantity of functional SERT protein in the cytoplasmic membrane of PASMC, the transporter activity seems to influence the uptake of serotonin [6]. Furthermore, risk of developing PH and its severity in the course of COPD appears to be associated with certain SERT gene polymorphisms, especially with the LL genotype of its promoter region [37, 38]. Accumulating evidence suggests that 5-HT receptors regulate the SERT activity in the PASMC membrane leading to lower serotonin uptake and in turn higher extracellular serotonin levels available for 5-HT receptor activation [30, 33].

In contrast to the constricting action of serotonin on PASMC, which is mainly mediated by 5-HT_1B_ receptors, the proliferative effects of serotonin require its internalization by the SERT [4, 34]. Serotonin that is taken up by the SERT activates not only mitogen-activated protein kinases representing proliferative stimuli, but also activates nicotinamide adenine dinucleotide phosphate (NADPH) oxidases leading to increased generation of reactive oxygen species (ROS), such as hydrogen peroxide. The subunit of NADPH oxidases, p22phox, is believed to play a role as a hypoxia sensor for HPV. In hypoxemic COPD patients, p22phox positively correlates with PAPm and oxygenation ratio as well as negatively with gas exchange capacity, suggesting that NADPH oxidases may facilitate HPV [39]. In COPD patients with or without PH, we found that the quantity of SERT availability, which can be considered as a surrogate for the quantity of internalized serotonin and thus potential inducer of NADPH oxidases (p22phox), did not correlate with oxygenation ratio or gas exchange capacity (DLCO). But it positively correlated with severity of PH (PVR), right heart strain (NT-proBNP), and airflow limitation (Fig. 4, Table 2), indicating a potential pathophysiologic link between pulmonary SERT availability, NADPH oxidases, ROS production, and thus HPV in COPD patients with or without PH.

Limitations of this study comprise the low number of participants, since for thorough analysis of effect size, a larger population will certainly be needed. Even though the gender distribution between the COPD cohorts including patients with or without PH was balanced (4 men, 1 woman, respectively), the control groups showed a different distribution (exclusively women in the PAH cohort, 2 men and 3 women in the HC). From these distribution differences, we cannot exclude the influence of gender-specific differences when comparing COPD cohort with PAH or healthy cohorts. Furthermore, we did not apply a compartment model for estimating binding potential using kinetic modelling of the [^11^C]DASB. We applied a semiquantitative approach to measure pulmonary [^11^C]DASB uptake by using the tissue-to-blood-concentration ratio after 25 to 30 min of tracer injection. We were aware that when selecting a time frame of interest, this should coincide with the transient equilibrium of the tracer in all subjects [17]. The HC did not undergo RHC, blood gas analysis, and exercise capacity.

In conclusion, pulmonary SERT availability was demonstrated using [^11^C]DASB and PET/CT in COPD patients with or without PH, in PAH patients as well as in HC. COPD patients, regardless of the presence of PH, showed reduced [^11^C]DASB uptake, while COPD patients who developed PH were found increased [^11^C]DASB uptake. These findings suggest that pulmonary SERT availability may reflect an important aspect in the pathogenesis of PH in patients with COPD, and its measurement may form the basis for further developments in the diagnosis and management of PH in COPD patients.

## Electronic supplementary material

ESM 1 (DOCX 331 kb)
