# Supplementary material for: Increased pulmonary serotonin transporter in patients with chronic obstructive pulmonary disease who developed pulmonary hypertension
Source: Eur J Nucl Med Mol Imaging. 2020 Oct 3;48(4):1081–92. doi: 10.1007/s00259-020-05056-7 (PMC8041706; doi:10.1007/s00259-020-05056-7)
Supplement: Supplementary file 1 — (DOCX 331 kb) [file 259_2020_5056_MOESM1_ESM.docx]

**Electronic Supplementary Material for**

**Increased pulmonary serotonin transporter in patients with chronic obstructive pulmonary disease who developed pulmonary hypertension**

**European Journal of Nuclear Medicine and Molecular Imaging**

Authors:

Armin Frille^1,2,^*, Michael Rullmann^2,3,^*, Georg-Alexander Becker^3^, Marianne Patt^3^, Julia Luthardt^3^, Solveig Tiepolt^3^, Hubert Wirtz^1^, Osama Sabri^3^, Swen Hesse^2,3,^*, Hans-Juergen Seyfarth^1,^*

^1^Department of Respiratory Medicine, University Hospital Leipzig, 04103 Leipzig, Germany

^2^Integrated Research and Treatment Center (IFB) Adiposity Diseases, University Medical Center Leipzig, 04013 Leipzig, Germany

^3^Department of Nuclear Medicine, University Hospital Leipzig, 04103 Leipzig, Germany

*These authors contributed equally to this work.

Corresponding author:

Prof. Dr. Hans-Juergen Seyfarth

Department of Respiratory Medicine, University of Leipzig, Liebigstrasse 20, 04103 Leipzig, Germany

Email: hans-juergen.seyfarth@medizin.uni-leipzig.de

**Supplementary Fig. 1** Clinical, hemodynamic and laboratory characteristics of participants. (a) Results from pulmonary function test and exercise capacity and (b) results from hemodynamic and biochemical assessment are shown. The figure legend applies to both panels. Group differences are shown as mean ± standard deviation and were calculated by using one-way ANOVA followed by Tukey’s post hoc correction (*P <0.05, **P <0.01, ***P <0.001), statistically non-significant comparisons are not labelled. *6MWD* 6 minute walking distance, *ANOVA* analysis of variance, *CI* cardiac index, *COPD* chronic obstructive pulmonary disease, *DLCO* diffusion capacity for carbon monoxide after single breath, *FEV_1_* forced expiratory volume in 1 s, *FVC* forced vital capacity, *HC* healthy control, *NT‑proBNP* N-terminal pro-brain natriuretic peptide, PAH pulmonary arterial hypertension, *PAPm* mean pulmonary arterial pressure, *PH* pulmonary hypertension, *PVR* pulmonary vascular resistance, *R_tot_* total airway resistance, *RV* residual volume, *TLC* total lung capacity

**Supplementary Fig. 2** Heat map of a correlation matrix comprising clinical, hemodynamic and laboratory characteristics of COPD patients with and without PH (COPD±PH). Results derive from Spearman rank correlation analyses where the coefficient rho is shown in the boxes and in the double gradient legend on the figure’s right side. For reasons of clarity, statistical significance was accepted at a level of a two-sided P <0.05 and is marked with an asterisk, whereas statistically not significant correlations are shown in light-coloured boxes. Lung tissue attenuation values result from a volume of interest in the middle lobe by means of computed tomography. *6MWD* 6 minute walking distance, *A-aO_2_* alveolar–arterial gradient, *CI* cardiac index, *DLCO* diffusion capacity for carbon monoxide, *FEV_1_* forced expiratory volume in 1 s, *FVC* forced vital capacity, *NT-proBNP* N-terminal pro-brain natriuretic peptide, *PAH* pulmonary arterial hypertension, *PaO_2_/FiO_2_* oxygenation ratio: arterial oxygen tension/inspiratory oxygen fraction, *PaCO_2_* arterial carbon dioxide tension, *PAPm* mean pulmonary arterial pressure, *pH* decimal logarithm of the reciprocal of the hydrogen ion activity, *PVR* pulmonary vascular resistance, *R_tot_* total airway resistance, *RV* residual volume, *SaO_2_* arterial oxygen saturation, *TLC* total lung capacity
